# Recognizing a Silent Threat: Canine Ehrlichiosis and Rickettsiosis in Northern Portugal

**DOI:** 10.3390/pathogens13121040

**Published:** 2024-11-27

**Authors:** Zbigniew Zając, Alejandro Cabezas-Cruz

**Affiliations:** 1 Department of Biology and Parasitology, Medical University of Lublin, Radziwiłłowska 11 St., 20-080 Lublin, Poland; 2 ANSES, INRAE, Ecole Nationale Vétérinaire d’Alfort, UMR BIPAR, Laboratoire de Santé Animale, F-94700 Maisons-Alfort, France

Vector-borne diseases pose significant challenges for both animal and public health worldwide. Among these, infections caused by *Ehrlichia canis* and *Rickettsia conorii* are of considerable importance [1]. *E. canis*, the causative agent of canine monocytic ehrlichiosis, can lead to severe clinical manifestations in dogs, including anemia, thrombocytopenia, and immunosuppression [2,3]. Meanwhile, *R. conorii*, responsible for Mediterranean spotted fever, represents a zoonotic pathogen with potentially severe outcomes in humans [4]. Dogs play a dual role as both hosts and reservoirs of these pathogens, underscoring their significance in the epidemiology of these diseases and the need for integrated control measures at the interface of animal and human health [5].

A recent cross-sectional study in Northern Portugal has provided concerning insights into the seroprevalence of *E. canis* and *R. conorii* among shelter dogs [6], underscoring a broader public health issue that often remains undetected. The study, leveraging both an enzyme-linked immunosorbent assay (ELISA) and an indirect immunofluorescence antibody test (IFAT), revealed a seroprevalence of 0.9% for *E. canis* and 9.7% for *R. conorii* among 113 dogs sampled from two different shelters.

Shelter environments, often characterized by high-density populations and limited resources for veterinary care, can facilitate pathogen transmission through ticks. This poses a potential zoonotic risk to shelter workers, volunteers, and future pet owners. Therefore, this study is pivotal in terms of not only its direct findings, but also their implications for the control and prevention of vector-borne diseases in domestic animals and, by extension, in human populations. The incidence of these diseases, facilitated by vectors such as ticks [7], is significant due to the vectors’ role in the transmission of pathogens, which can lead to severe and sometimes fatal conditions in both animals and humans [5,8].

The demographic findings were also explored, where higher odds ratios of *R. conorii* seropositivity were found among female dogs, and there were higher instances of co-infection in one of the shelters, highlighting the nuanced dynamics of disease prevalence based on geographical and biological variables. These insights are crucial for veterinary public health officials to develop targeted interventions that could mitigate the risk of widespread disease transmission.

Moreover, this study’s focus on shelter dogs—a population often overlooked in veterinary research—amplifies its importance. Shelter dogs can serve as sentinels for human health given their higher exposure rates to disease vectors and their frequent proximity to humans [9]. This study thereby not only charts the prevalence of these diseases, but also sets a precedent for future epidemiological studies and the need for a robust One Health approach.

This One Health approach would see the convergence of veterinary science, medical science, and environmental science to forge pathways that could lead to better health outcomes across species. This is particularly pertinent given the ease with which diseases can spread in our increasingly interconnected world—a risk exacerbated by global travel and climate change influencing vector populations and behaviors.

Thus, while this study focused on a specific regional context, its implications resonate on a global scale, necessitating a unified approach to health and disease management. The work performed by the researchers not only adds a significant piece to the epidemiological puzzle, but also serves as a call to action for public health officials to consider novel strategies in disease monitoring and control. To mitigate these risks, the authors propose several critical measures: routine serological screening of shelter dogs, the implementation of effective vector control strategies, and the education of shelter personnel about zoonotic risks. These interventions can significantly reduce the prevalence of *E. canis* and *R. conorii* infections in dogs and minimize the likelihood of transmission to humans. Additionally, expanded research into the epidemiology of these pathogens across diverse geographic regions is essential to establishing a comprehensive understanding of their dynamics and informing effective prevention strategies.

In conclusion, this study highlights the critical role of shelter dogs in the transmission ecology of *E. canis* and *R. conorii*. Addressing these infections in canine populations is vital for improving animal welfare and protecting public health, aligning with the principles of the One Health approach. By adopting proactive surveillance and prevention strategies, and by fostering inter-disciplinary collaboration, we can better anticipate and mitigate the impacts of zoonotic diseases, protecting both animal and human health. This study is a testament to the critical need for ongoing research and dialog within the scientific community and between the public and policy makers to ensure a safe and healthy future for all.
